# Highly Sensitive MXene/MMT-Based Hydrogel for Wearable Sensors and Flexible Supercapacitors

**DOI:** 10.3390/gels11121000

**Published:** 2025-12-11

**Authors:** Haiyan Zhao, Ziqi Wang, Chaohao Yin, Chu Chen, Li Wang, Xin Zhang, Zhuo Wang

**Affiliations:** 1 Liaoning Key Laboratory of Plasma Technology, School of Physics and Materials Engineering, Dalian Minzu University, Dalian 116600, China; 2 College of Resources and Environment, University of Chinese Academy of Sciences, Beijing 101408, China; 3 Beijing Institute of Nanoenergy and Nanosystems, Chinese Academy of Sciences, Beijing 101400, China

**Keywords:** conductive hydrogel, supercapacitor, wearable, sensor, physical signal

## Abstract

In this work, a flexible, stretchable, tough, highly ionic conductive, and anti-freezing hydrogel based on acrylamide/two-dimensional transition metal carbide (MXene)/montmorillonite (MMT) was precisely designed. In the hydrogel, MXene and MMT acted as both cross-linking agents and conductive fillers, delivering high stretchability (1037%) with a strength of up to approximately 67 kPa and high conductivity. As a result, the usual trade-off between conductivity and mechanical properties of hydrogels could be alleviated to some extent. Therefore, the hydrogel could be used as an electrolyte for supercapacitors (SCs) and strain sensors to monitor physical signals. The hydrogel-based SC exhibited outstanding electrochemical performance over a wide temperature range. Moreover, it could easily withstand various deformations, such as bending, twisting, and compression. The hydrogel also exhibited excellent sensing properties, with a short tensile response time and a high-sensitivity factor (GF = 14.8) in the 0–400% range (0 denotes the original state, where both the strain and stretch are zero as there is no deformation at this point). Due to its high conductivity, the prepared hydrogel could be used as a flexible electrode to replace commercial electrodes and record electromyographic (EMG) signals. This work proposes a novel approach for balancing the conductivity and mechanical strength of hydrogels.

## 1. Introduction

Flexible electronics capable of functioning under deformation and withstanding bending, rolling, and twisting [1], have gained widespread attention in recent years for their potential in wearable devices, health monitoring, soft robotics, and environmental applications [2,3,4]. Various candidate materials have been investigated based on the requirements of flexible electronics [5,6]. In particular, conductive hydrogels have been widely studied as promising materials for developing flexible electronics due to their high-performance and easy fabrication processes. The stretchability, biocompatibility, and conductivity make them potential candidates for sensors [7], supercapacitors (SCs) [8,9], actuators [10], adhesive implant–tissue interfaces [11], etc. However, conventional conductive hydrogels inevitably freeze at subzero temperatures, significantly impeding ionic transport. So, the hydrogels cannot preserve their conductivity and mechanical properties, which severely limits their practical applications in low-temperature environments [2,12,13,14,15]. Further, water in hydrogels evaporates at room temperature, which impedes their durability and stability [2,16]. Hence, it is highly desirable to develop highly conductive hydrogels with anti-freezing properties, long-lasting moisture resistance, and long-term stability for flexible electronic devices.

Due to their high conductivity, two-dimensional (2D) transition metal carbides/nitrides (MXenes) have recently gained significant attention. However, they tend to aggregate inhomogeneously, deteriorating their intrinsic properties. It is believed that inserting a nanospacer between intensively stacked MXene nanosheets can effectively expand their interlaminar space [17], which helps improve the electrode and guarantees ion transportation between interlayers. Moreover, it is also a promising photothermal agent for potential applications in photothermal therapy (PTT) [18]. It is widely accepted that the interaction between MMT and MXene enables MMT to function as a nanospacer [19]. Considering the above, montmorillonite (MMT), with abundant functional groups (-COOH, -OH, and Ca^2+^), can significantly facilitate bonding between MMT, MXene, and polymer chains to enhance the mechanical strength of the hydrogel. In addition, the positive effects of MMT and Mxene on conductivity were recently reported [20].

Herein, we report a novel multifunctional organohydrogel formed by in situ polymerizing acrylamide within a network co-crosslinked by MXene and MMT, using a glycerol–water binary solvent. The strategy conveys the following advantages: First, MXene and MMT act not only as conductive fillers and mechanical reinforcers, respectively, but also collaboratively as dynamic cross-linkers. The abundant functional groups on MXene (e.g., -O, -OH) and the ionic sites on MMT form extensive hydrogen and ionic bonds with the PAM chains, constructing a hierarchical network that effectively alleviates the classic conductivity–mechanical property trade-off. Second, we provide mechanistic evidence that MMT serves as an effective nanospacer to impede MXene restacking, expanding the interlayer spacing and thereby facilitating enhanced ion transport kinetics, which is critical for high-performance supercapacitors. Third, the incorporation of the glycerol–water binary solvent endows the hydrogel with remarkable anti-freezing capability (down to −20 °C) and long-term moisture retention, significantly broadening its operational environment. Finally, beyond conventional sensing and energy storage, we exploit the intrinsic photothermal property of MXene for potential light-responsive applications and demonstrate the hydrogel’s versatility as a unified platform for high-sensitivity strain/pressure sensors, all-temperature flexible supercapacitors, reliable bio-electrodes for electrophysiological signal recording (e.g., EMG), and even preliminary exploration in human–machine interaction. Therefore, this work presents a high-performance material and explores the multifunctional properties required for next-generation flexible and wearable electronics.

## 2. Results and Discussion

**Scheme 1:**
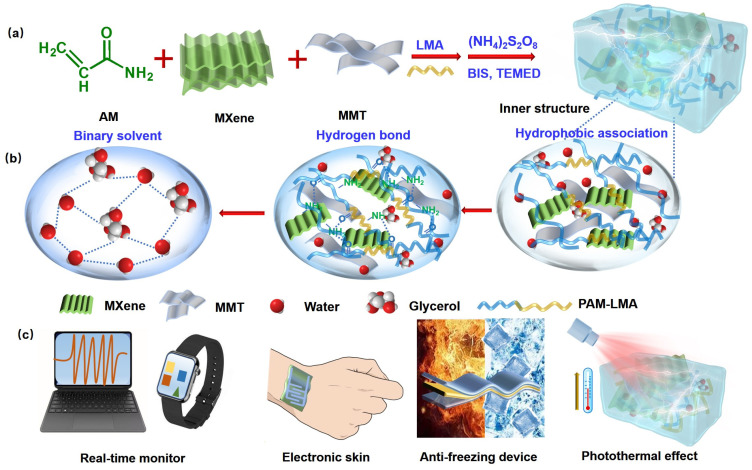
Schematic diagram of the fabrication process, chemical structure, and application of the PAMMXLL hydrogel. (**a**) The fabrication process of the PAMMXLL hydrogel; (**b**) schematic diagram of the inner chemical structure of the hydrogel; and (**c**) applications of the PAMMXLL hydrogel.

Scheme 1a illustrates the synthesis, structure, and application of flexible and conductive hydrogels. In this concept, lauryl methacrylate (LMA) and AM were copolymerized using ammonium polysulfate (APS) and N,N,N′,N′-tetramethylethylenediamine (TEMED) as redox initiators, where the hydrophobic association between PAM and LMA formed a dynamic cross-linking network as reported [19]. Meanwhile, MXene and MMT acted as conductive fillers and cross-linkers because of the abundant functional groups in MXene and the ions in MMT. Carboxyl, amidogen, and halogen in MXene could bond with AM and MMT to form hydrogen bonds (Scheme 1b). Meanwhile, MMT ions could bond with carboxyl groups of AM or MXene through covalent and ionic bonds. So, the entangled network structure in the hydrogel reinforced the mechanical properties of the hydrogel. At the same time, the conductivity of MXene and MMT endowed the hydrogel with high conductivity. Therefore, by this method, the addition of the conductive filler guaranteed both the conductivity and the mechanical properties of the hydrogel, alleviating the trade-off between conductivity and strength. In addition, the binary solution of Gly and H_2_O enabled the hydrogel to have a wide range of operating temperatures. Moreover, MXenes have recently gained significant attention as promising photothermal agents for PTT potential applications. So, the above characteristics make the hydrogel a potential candidate for real-time monitoring, SCs, and other applications (Scheme 1c).

**Figure 1:**
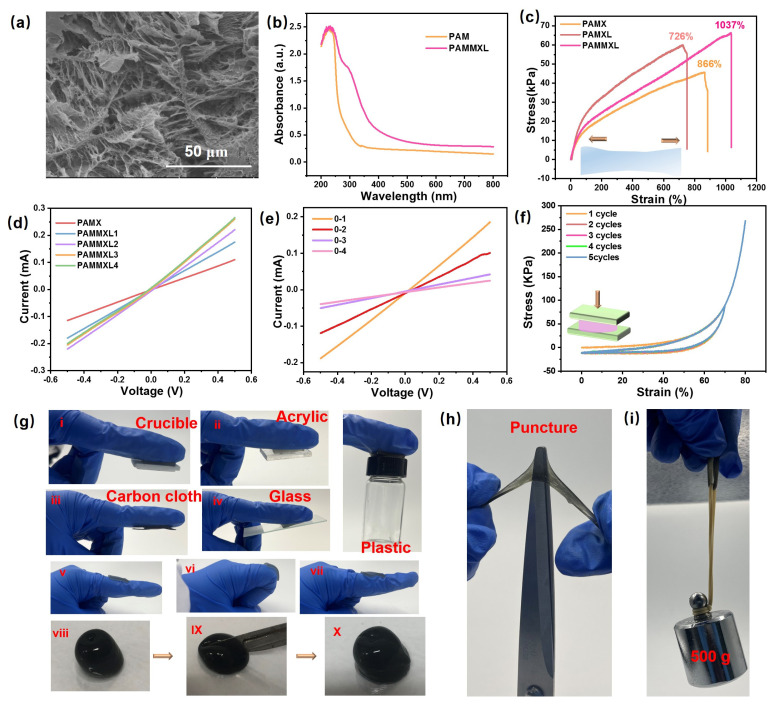
Characteristics of the PAMMXLL hydrogel. (**a**) The SEM image of the hydrogel. (**b**) The UV absorption spectrum. (**c**) The tensile stress–strain curves of the hydrogels. (**d**) The LSV curves of the PAMMXLL with different applied strains. (**e**) The LSV curves of the hydrogels with different amounts of MMT. (**f**) The fatigue resistance of an original and recovered PAMMXLL sample. (**g**) Photographs showing the adhesive properties of the PAMMXLL, hydrogel (**i**) crucible (**ii**) acrylic (**iii**) carbon cloth (**iv**) glass (**v**–**vii**) bending finger (**viii**–**x**) recovered feature of the hydrogel. (**h**,**i**) Mechanical performance of PAMMXL when stabbed with a shear and used to lift a weight plate.

The inner structure of the hydrogel was confirmed by SEM micrographs (Figure 1a). The hydrogel comprised a three-dimensional (3D) network with a pore size of around 25 μm × 5 μm. The ultraviolet (UV) absorption of PAMMXL was significantly enhanced compared with PAM, which is attributed to the doping of MXene (Figure 1b). Then, the effect of MMT and LAM on the mechanical properties of the hydrogel was investigated (Figure 1c). The addition of MMT was beneficial for the strength of the hydrogel. Impressively, the addition of LMA simultaneously enhanced the strength and the stretchability of the hydrogel. This confirmed the positive role of MMT and LMA in enhancing the mechanical properties of the hydrogel. After that, the effects of the amount of MMT on conductivity were investigated in detail. As shown in Figure 1d, the conductivity was further enhanced after adding MMT, but it did not increase monotonously. The conductivity first increased with the MMT amounts 0.01 g (PAMMXLL1), 0.02 g (PAMMXLL2), and 0.03 g (PAMMXLL3, PAMMXLL). However, the conductivity did not change when the MMT amount was further increased to 0.05 g (PAMMXLL4). So, PAMMXL3 was chosen for further investigations, which was noted as PAMMXLL. Then, the conductivity of the hydrogel with different stretching states was explored, as given in Figure 1e. The conductivity decreased with stretching from 0 to 4 cm. Subsequently, a group of tensile tests was carried out, as shown in Figure 1f. The hysteresis loops nearly remained constant during the five tensile cycles. This indicates that PAMMXLL could sustain its interior network structure during the test. The synergistic effect of MMT and LMA led to excellent robust elasticity and self-recovery properties for PAMMXLL. Self-adhesiveness is vital for the interface connection of the hydrogel [21]. Because of the abundant functional groups in the hydrogel, this hydrogel could adhere to a wide range of surfaces, such as crucibles, acrylic, carbon cloth, glasses, and plastics, as demonstrated in Figure 1g. More significantly, the self-adhesive PAMMXL exhibited excellent shape adaptability, and it could directly attach to irregular surfaces, such as a finger, and withstand the bending of the finger joint (Figure 1g(v–vii)). In addition, PAMMXLL was confirmed to endure damage from sharp objects. When the PAMMXLL hydrogel was treated with a sharp knife, it could be recovered to its original state instantly without visible breakage or scars (Figure 1g(viii–x)), and it also showed puncture resistance (Figure 1h). The PAMMXLL hydrogel could also lift a weight of 500 g, exhibiting excellent toughness and strength (Figure 1i).

**Figure 2:**
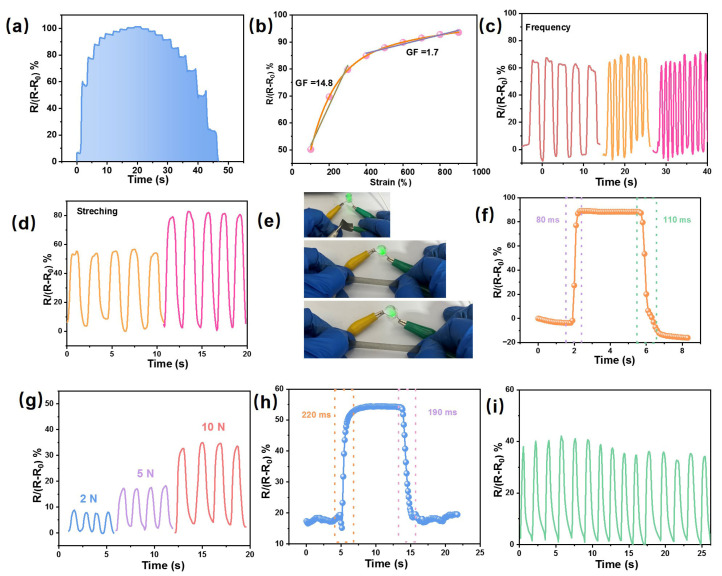
(**a**) ΔR/R_0_ variation in the PAMMXLL hydrogel under stretching and releasing from 0 to 900% strains. (**b**) ΔR/R_0_ and calculated GF value of the PAMMXLL sensor under different applied strains. (**c**) Changes in the resistance of the PAMMXLL sensor when stretched to 100% at different stretching speeds (25, 35, and 40 cycles/min). (**d**) Five stretching and releasing cycle tests of the hydrogel strain sensor at strains of 100−200%. (**e**) Photographs of changes in brightness of the LED at different hydrogel elongations (0%, 300%, and 500%). (**f**) Response time and recovery time of the PAMMXLL hydrogel at strain of 300%. (**g**) Four compressing and releasing cycle tests of PAMMXLL in the pressure range of 2–10 N. (**h**) Response time and recovery time of the PAMMXLL pressure sensor. (**i**) Fifteen cycle stability test in the compressed state.

The curve of resistance changes versus strain is shown in Figure 2a. The curve can be divided into two linear regions, with corresponding gauge factors (GFs) of 14.8 (0–300%) and 1.7 (400–900%), respectively (Figure 2b). The change in ΔR/R_0_ is an important characteristic for wearable device applications [22]. The hydrogel showed a negligible ΔR/R_0_ change during cycles at a strain of 100% with stretching speeds from 25 to 40 cycles/min (Figure 2c). Furthermore, the hydrogel strain sensor showed a rapid electrical response at different strain states, as shown in Figure 2d, which was due to the reversible dynamic physical cross-linking network in the hydrogel. The high conductivity of PAMMXLL hydrogel enabled it to power a light-emitting diode (LED) connected to an electrical circuit during elongation (Figure 2e). Obviously, the LED remained lit even when the elongation of the hydrogel varied from 0% to 500%, which confirms the high conductivity of the hydrogel. It is impressive that the PAMMXL sensor showed an ultrafast response (80 ms) and a short recovery time (110 ms) without electrical hysteresis during tensile tests, which are commendable characteristics for strain sensors (Figure 2f). Apart from strain sensing, this PAMMXL hydrogel sensor exhibited sensitive properties toward different compression levels. Moreover, the sensor showed good reproducibility of pressure response with a fast response (220 ms) and a short recovery time (190 ms) (Figure 2g,h). This indicates that the hydrogel could act as both a tensile and compression sensor. These results indicate that ΔR/R_0_ is independent of tensile or compression rate. The hydrogel showed decent stability as a compression sensor (Figure 2i).

**Figure 3:**
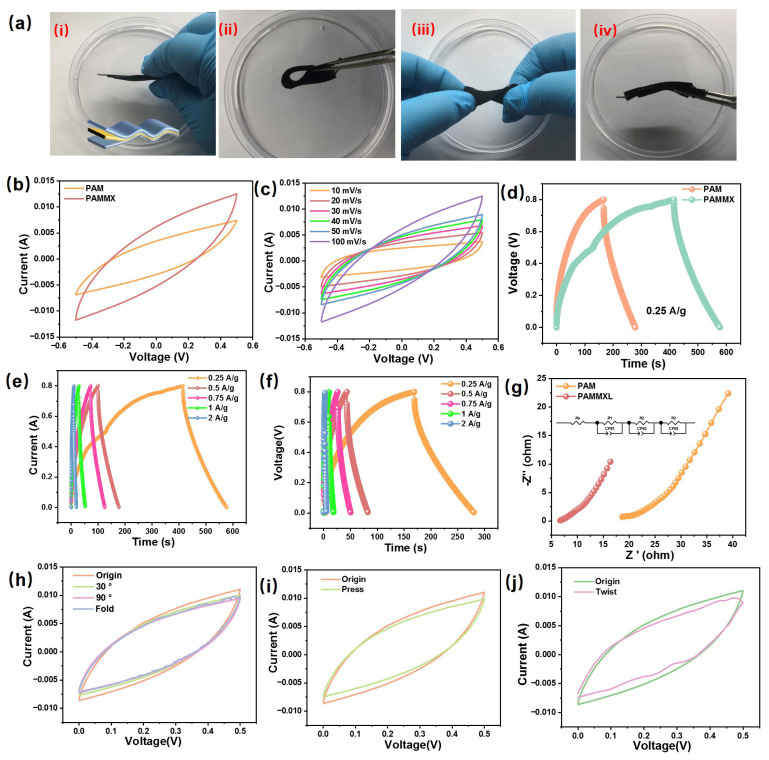
(**a**) Photographs of PAMMXL-based SC at different states: (**i**) original, (**ii**) bending, and (**iii**) twisting, and (**iv**) recovered after bending and twisting. (**b**) The comparison of CV curves of PAM and PAMMXL hydrogels as electrolytes for SCs. (**c**) The CV curves of PAMMXL-based SC at different sweeping rates. (**d**) The comparison of the GCD curves of PAM and PAMMXL hydrogels as electrolytes for SCs. (**e**,**f**) The GCD curves of PAMMXL- and PAM-based SC at different current densities under a voltage of 0.8 V. (**g**) The EIS data of the PAM and PAMMXL hydrogel-based SCs in the frequency range from 0.01 Hz to 100 kHz, the inset shows the equivalent circuit based on the EIS results. (**g**) The comparison of the capacitance of PAM and PAMMXL hydrogels as electrolytes for SCs at different current densities with five cycles. (**h**–**j**) The CV curves of PAMMXL-based SC with bending (**h**), twisting (**i**), and pressing (**j**).

The high conductivity and self-adhesion of the PAMMXL hydrogel made it a suitable electrolyte for SCs. As shown in Figure 3a, the SC was fabricated with the as-synthesized hydrogel as the electrolyte and MCNT-coated carbon cloth as electrodes. The adhesive character of the hydrogel ensured a tight fit between the electrode materials and the electrolyte, which is essential for SCs to withstand deformation and ion transportation. Figure 3a shows that the fabricated PAMMXL hydrogel-based SC (PAMSC) could endure bending and twisting without separating the electrode and electrolyte. The cyclic voltammetry (CV) curve of the PAMMXL hydrogel-based SC showed a higher integrated surface area compared to the PAM hydrogel-based SC (Figure 3b and Figure S2). The CV results for the PAMMXL hydrogel-based SC showed a nearly rectangular profile from 10 to 100 mV s^−1^, indicating an ideal double-layer capacitance behavior, excellent capacitive performance, and rate capability [23] (Figure 3c). Moreover, galvanostatic charge and discharge (GCD) was employed to evaluate electrochemical capacitance. Accordingly, the GCD profiles of the PAM and PAMMXLL hydrogel-based SCs were measured at a current density of 0.5 A g^−1^ with operational voltages ranging from 0 to 0.8 V, as given in Figure 3d–f. The GCD curve of the SC resembled a triangle with negligible voltage drop, and the PAMMXL hydrogel-based SC had a longer discharging time. In addition, the capacitance of the PAMMXL hydrogel-based SC was higher than that of the PAM hydrogel-based SC (Figure 3d). Subsequently, the galvanostatic charge–discharge (GCD) processes of the PAM and PAMMXL hydrogel-based supercapacitor (SC) were measured at various current densities (0.25–2 A g^−1^), as depicted in Figure 3e,f. The GCD profiles exhibited nearly symmetrical triangular shapes, confirming highly reversible charge–discharge behavior at the electrode–electrolyte interface. The capacitances were around 130 F g^−1^ and 53 F g^−1^ for PAMML and PAM hydrogels, respectively, at a current density of 0.25 A g^−1^. A comparison of capacitance of PAM and PAMMXLL hydrogels as electrolytes for SCs at different current densities is shown in Figure S3. The PAMMXL hydrogel-based SC consistently showed much higher capacitance compared with PAM. Then, the electrochemical impedance spectroscopy (EIS) measurements were performed using PAM and PAMMXL hydrogels as electrolytes at open circuit voltage (V_OC_) (Figure 3g). As shown in Figure 3g, the PAMMXL hydrogel-based SC showed a higher conductivity compared with PAM hydrogel-based SC. The inset gives the equivalent circuit based on the EIS results, which can be divided into three parts: high-frequency region, which typically corresponds to surface film processes; medium-frequency region, which usually corresponds to charge transfer processes; and low-frequency region, which generally corresponds to mass transfer/diffusion processes (or deeper interfacial reactions), where R refers to “diffusion-related resistance”. As wearable devices, SCs should be able to endure external forces, such as bending, twisting, and pressing. So, the PAMMXL hydrogel-based SC was fabricated, and its performance towards external forces was recorded, as shown in Figure 3h–j. The CV curves of the SC under bending, pressing, and twisting showed that it nearly maintained its pristine shape in all states, confirming that it can be used as a wearable electronic device. In addition, the stability of the SC was investigated, as shown in Figure S4. It possessed relatively satisfactory electrochemical stability, with 86% capacitance retention over 1000 cycles at a sweep rate of 100 mV^−1^.

**Figure 4:**
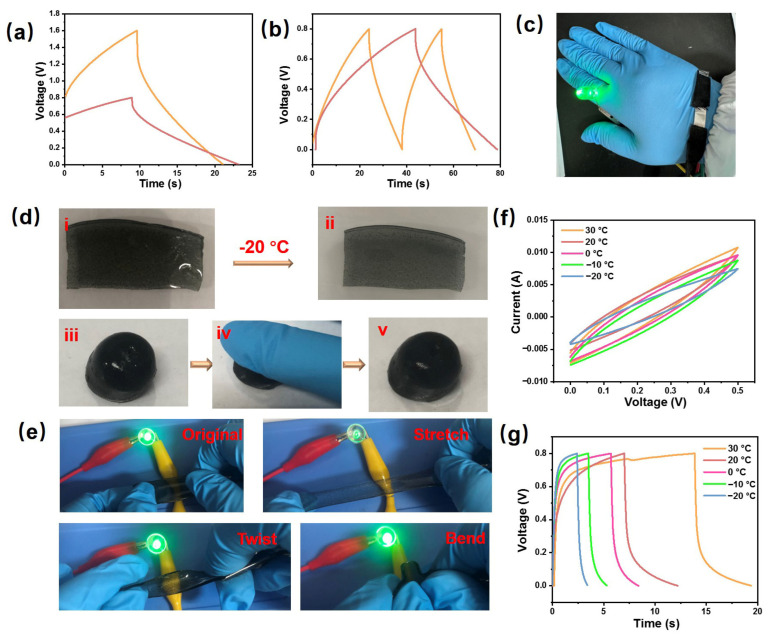
(**a**,**b**) GCD curves of single, two series (**a**), and two parallel (**b**). (**c**) A photograph of the wearable SCs in series to power a commercial LED lamp. (**d**) Photographs of the PAMMXL hydrogel at −20 °C with pressing (**i**–**v**). (**e**) The PAMMXL hydrogel as a conductor to enlighten LED at different states at −20 °C. (**f**) The CV curves of the device under different operation temperatures. (**g**) The GCD curves of the device at 2 A g^−1^ under different operation temperatures.

The flexible SCs were connected in series or in parallel to reach a higher voltage or current and prove the practical applications of the SC. As shown in Figure 4a, the potential could be tailored with the number of SCs in series, and the working voltage of the operation potential increased from 0.8 to 1.6 V. In addition, the stored charge of two SCs in parallel was two times that of the single one at the same current density (Figure 4b). So, as shown in Figure 4c, this integrated SC could enlighten a commercial LED lamp, demonstrating the practical application of the SC as a wearable device. In addition, the operation temperature range is a critical parameter for flexible SCs, since the devices should properly function under low temperatures. In this hydrogel platform, H_2_O and Gly were used as binary solutions, and abundant hydrogen bonds among Gly, PAM, and MXene were formed to ensure a wide operation temperature. As demonstrated in Figure 4d, the hydrogel remained flexible at −20 °C and could be pressed and recovered to its original state, which confirms its anti-freezing properties. Furthermore, the hydrogel acted as a conductor and could enlighten an LED at −20 °C during stretching, bending, and twisting, indicating high conductivity at low temperatures (Figure 4e). Then, the CV and GCD performance were tested over the temperature range from −20 to 30 °C, and the test showed good cycling stability (Figure 4f,g). These features showed the great potential of SCs for practical applications.

**Figure 5:**
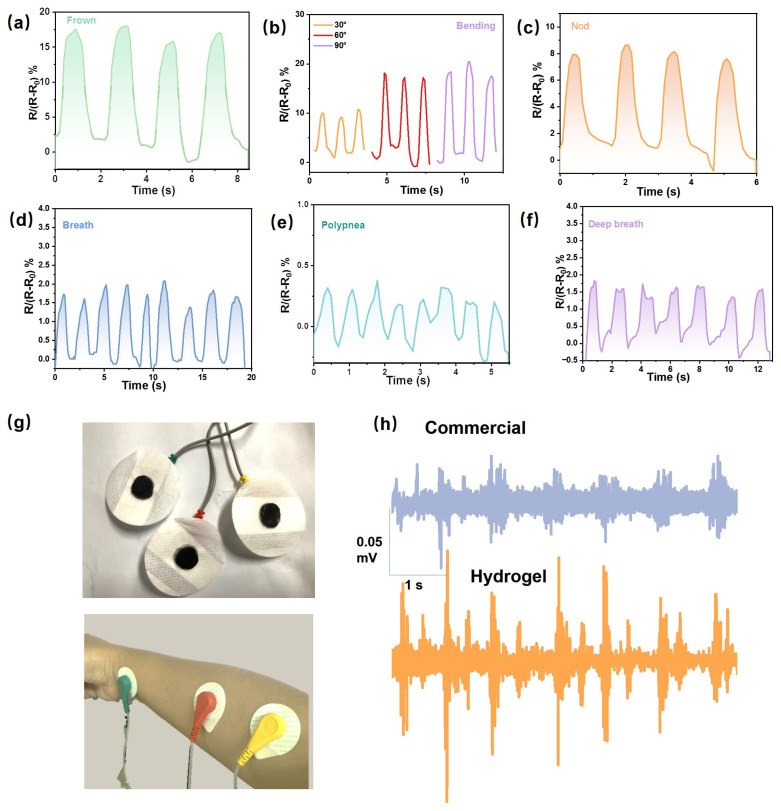
(**a**–**f**) Changes in resistance for the PAMMXL hydrogel in response to (**a**) finger bending at the angles of 30°, 60°, and 90°, (**b**) frowning, (**c**) nodding, (**d**) normal breathing, (**e**) polypnea, and (**f**) deep breaths. (**g**) Photos of the hydrogel as an electrode for EMG. (**h**) The EMG signals of a volunteer. Comparison of a commercial electrode and the hydrogel as the electrode for measuring EMG signals from the forearm muscle.

The superior mechanical performance of the PAMMXL hydrogel enabled its use as a wearable sensor for biomonitoring. The hydrogel sensor was first strapped to a finger to record the change in the relative resistance of the bending finger (30°, 60°, and 90°), indicating that the sensor could detect different ranges of motions (Figure 5b). Then, the hydrogel sensor was attached between the eyebrows and neck, and the sensor demonstrated fast and sensitive responses to frowning and nodding, indicating that the sensor could distinguish tiny strains (Figure 5a,c). Furthermore, the sensor produced sensitive responses to breathing while fixed to the abdomen and could detect both normal and abnormal breathing of the volunteer. This indicates that the sensor could realize the detection of frequency and intensity, as given in Figure 5d–f. Moreover, when the stress was released, the resistance quickly fell back to its initial state, indicating sensitive, timely, and stable sensing. Due to the high conductivity and stretchability of the PAMMXL hydrogel, it could be fabricated as an alternative electrode material for recording electrophysiological signals such as electromyographic (EMG) signals. As shown in Figure 5g, the PAMMXL hydrogel acted as the connection and interface between intrinsically stretchable hydrogels and rigid metal elements. In this concept, three PAMMXL electrode pads were attached to the right arm and right ankle to record EMG signals, as shown in Figure 5h. Typically, compared with the commercial electrode for EMG, the PAMMXL hydrogel showed a similar waveform. The process of fist-clenching and releasing led to obvious changes in potential intensity. This indicates that the PAMMXL electrode could gather high-quality electrophysiological signals and has the potential for use as a flexible electrode in electronic devices.

**Figure 6:**
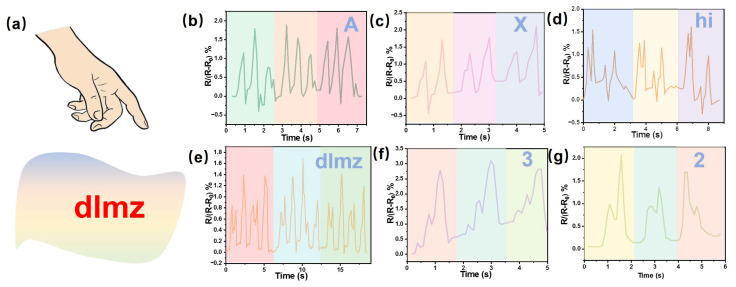
(**a**) The schematic diagram of the PAMMXL hydrogel as a sensor for writing. (**b**,**c**) Demonstration of the PAMMXL sensor in response to letters “A” and “X”. (**d**,**e**) Demonstration of the PAMMXL sensor for words “hi” and “dlmz” 3×. (**f**,**g**) Demonstration of the PAMMXL sensor for numbers “3” and “2” 3×.

Interestingly, as demonstrated in Figure 6a, the PAMMXL hydrogel-based sensor could also serve as a drawing board, allowing writing on it. It could accurately recognize letters, words, or numbers. For example, it could identify the letters “A” and “X” three times with repeated waveforms and consistent resistance variation (Figure 6b,c). Beyond letters, the PAMMXL-based sensor could distinguish words and numbers sensitively and steadily (Figure 6d–g). This indicates that the hydrogel has great potential in human–machine interactions.

**Figure 7:**
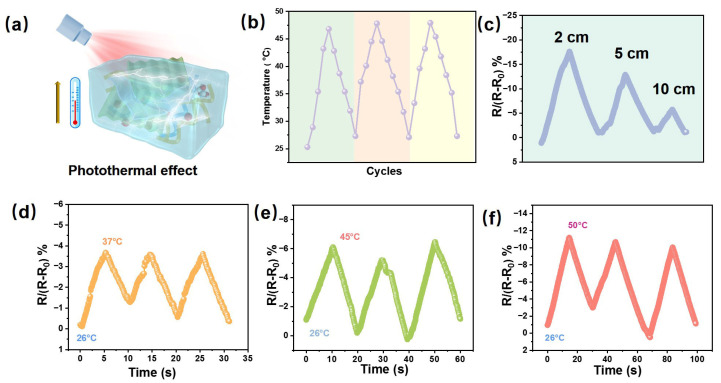
(**a**) The schematic diagram of photothermal conversion and temperature-sensitive properties of PAMMXL hydrogel. (**b**) Three cycles of surface temperature changes of the PAMMXL hydrogel under repeated NIR laser irradiation. (**c**) Continuous monitoring of the resistance of PAMMXL hydrogel under different NIR laser irradiation distances on the skin surface. (**d**–**f**) Continuous monitoring of the resistance of PAMMXL hydrogel under temperature fluctuations (26–37 °C, 26–45 °C, and 26–50 °C) on the skin surface.

The PAMMXL hydrogel also serves as an effective smart wearable sensor with significant photothermal conversion capabilities due to MXene. Under NIR laser irradiation (808 nm, 0.5 W cm^−2^), the material quickly generated heat, which could be applied in the field of wound repair (Figure 7a) [24,25]. This photothermal response remained consistent over repeated tests (Figure 7b). Beyond heating, PAMMXL also exhibited sensitive sensing functions. It could detect variations in NIR laser distance, as evidenced by a decrease in the relative resistance change (ΔR/R%) when the distance increased from 2 cm to 10 cm (Figure 7c). Moreover, when worn on the skin, PAMMXL accurately monitored temperature changes from 26 °C to 50 °C, with its resistance reliably responding to heating and cooling cycles (Figure 7d–f).

Although promising results were achieved, this study is not without limitations. The current performance evaluation, particularly concerning the achievable capacitance and rate capability, remains preliminary. The lack of detailed characterization limits the depth of mechanistic explanation offered. Therefore, the reported performance parameters should be interpreted within this context, highlighting an avenue for more extensive future research.

## 3. Conclusions

This study presents a novel conductive hydrogel (PAMMXL hydrogel) with high conductivity, self-adhesion, excellent sensitivity, and anti-freezing ability. The fabrication of PAMMXL hydrogel involved the polymerization of MXene and MMT in the PAM solution. The strong interactions between MXene and MMT in the hydrogel system led to high mechanical strength and even distribution of MXene. Based on these characteristics, it could be assembled as a multifunctional flexible sensor for monitoring human motions and as an electrolyte for SCs to store energy. Impressively, the 3D conductive network constructed by MXene and MMT endowed the sensor with excellent sensitivity (GF = 14.8) and even as a reliable bio-interface for recording electrophysiological signals. Furthermore, when used as an electrolyte for SCs, the hydrogel delivered high capacitance over a wide temperature range. This work offers a feasible and promising strategy for developing versatile hydrogels applicable to flexible wearable devices and electronic skins.

## 4. Materials and Methods

### 4.1. Materials

Mxene solution was purchased from Foshan Xinxi Technology, Co., Ltd., Foshan, China; MMT and lauryl methacrylate (LMA, 96%) were purchased from Maclin Technology Co., Ltd., Shanghai, China; acrylamide (AM) was purchased from Aladdin Biochemical Technology Co., Ltd., Shanghai, China. All chemicals were used without purification.

### 4.2. Preparation of the Hydrogels

The acrylamide (AM, 3 g) powder was first dissolved in a mixed solution of water and ethylene glycol (10 mL), with a water-to-ethylene glycol ratio of 7:3, under continuous magnetic stirring for 5 min at room temperature. Subsequently, 500 μL MXene (5 mg mL^−1^) and MMT were added to the AM solution after stirring and sonicating for one hour. Then, 20 μL lauryl methacrylate (LMA) was added to the system. Next, 0.12 g ammonium polysulfate (APS) and 0.003 g N,N′-methylenebisacrylamide (BIS) were added into the above solution and stirred for another 3 min. Finally, 20 μL of N,N,N′,N′-tetramethylethylenediamine (TMEDA) was injected into the reaction mixture. LMA and acrylamide (AM) were copolymerized using APS-TEMED [19]. Then, the above solution was transferred into a dish for further polymerization. The final products were named PAM, PAMX (0 g), PAMMXLL1 (0.01 g), PAMMXLL2 (0.02 mg), PAMMXLL3/PAMMXLL (0.03 g), and PAMMXLL5 (0.05 g) according to the amount of MMT.

### 4.3. Characterizations

The morphology and structure of the hydrogels were characterized using field-emission scanning electron microscopy (Nova NanoSEM 450, FEI, Hillsboro, OR, USA). The electrochemical performance of the hydrogel and SCs was analyzed using an electrochemical workstation (CHI 660E, Chenhua, Shanghai, China).

## Data Availability

The data that support the findings of this study are available from the corresponding author upon reasonable request. The data of this study have been made available within the paper.

## Supplementary Materials

The following supporting information can be downloaded at: https://www.mdpi.com/article/10.3390/gels11121000/s1, Figure S1: The comparison of the fabrication of hydrogels with (right) and without (left) MMT; Figure S2: The CV curves of PAMK hydrogel as electrolyte; Figure S3: The comparison of the capacitance of PAM and PAMMXL hydrogels as electrolytes for SCs at different current densities; Figure S4: The cycle life of the hydrogel during 1000 cycles at a scan rate of 100 mV s^−1^.
