# Supplementary material for: Highly Sensitive MXene/MMT-Based Hydrogel for Wearable Sensors and Flexible Supercapacitors
Source: Gels. 2025 Dec 11;11(12):1000. doi: 10.3390/gels11121000 (PMC12732765; doi:10.3390/gels11121000)
Supplement: Supplementary file 1 [file gels-11-01000-s001.zip › gels-3989636-supplementary.pdf]

Supporting Information

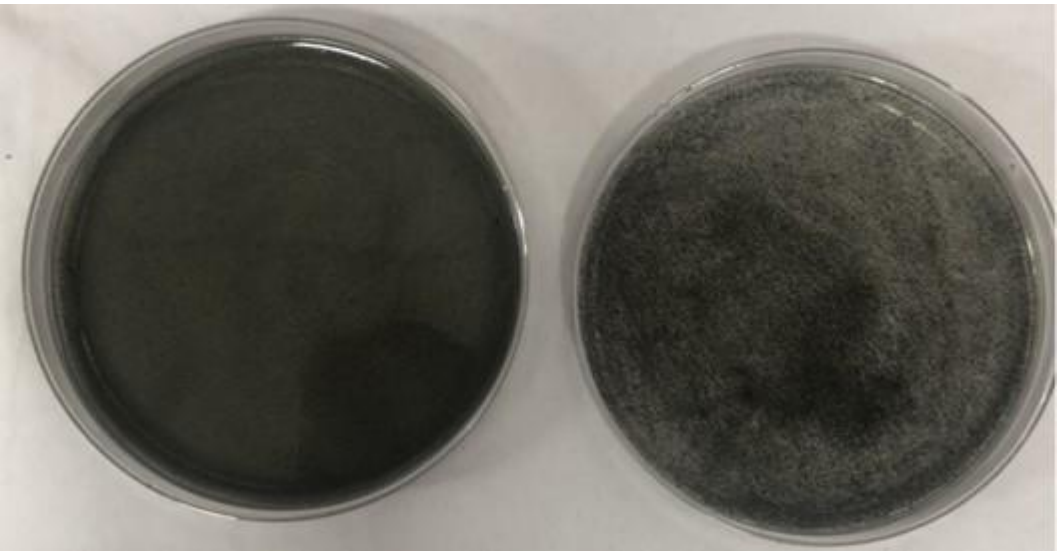

Figure S1. The comparison of the fabrication of hydrogels with (right) and without (left) MMT.

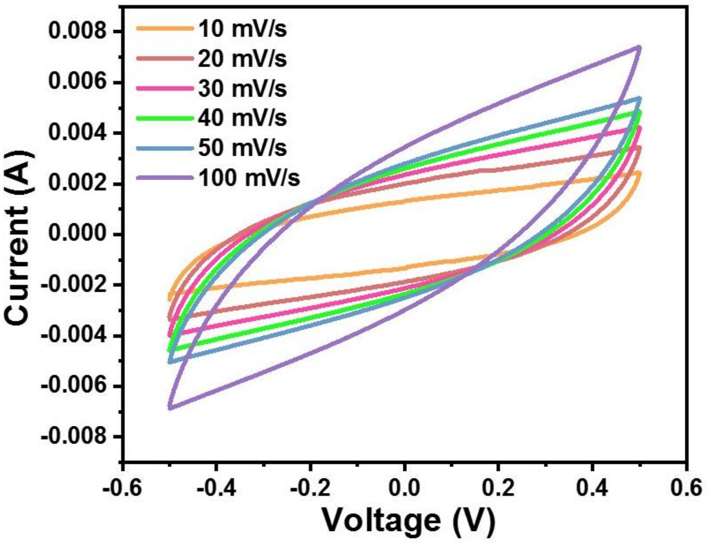

Figure S2. The CV curves of PAMK hydrogel as electrolyte.

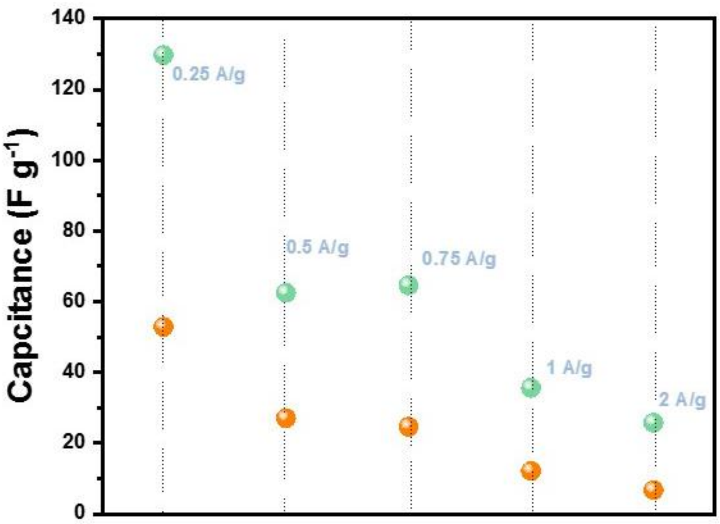

Figure S3. The comparison of the capacitance of PAM and PAMMXL hydrogels as electrolytes for SCs at different current densities.

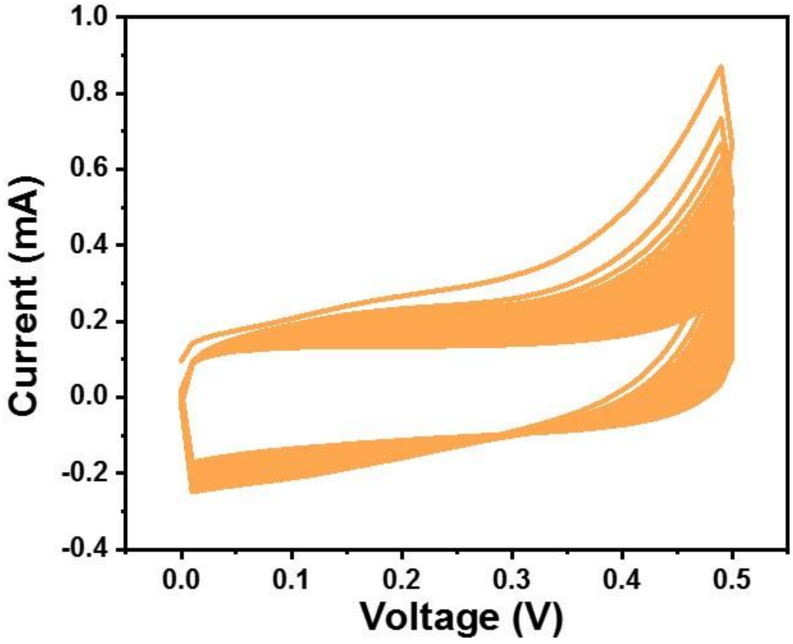

Figure S4. The cycle life of the hydrogel during 1000 cycles at a scan rate of 100 mV s^{-1}.
